# Considerations for Exchanging and Sharing Medical Images for Improved Collaboration and Patient Care: HIMSS-SIIM Collaborative White Paper

**DOI:** 10.1007/s10278-016-9885-x

**Published:** 2016-06-28

**Authors:** Amy Vreeland, Kenneth R. Persons, Henri (Rik) Primo, Matthew Bishop, Kimberley M. Garriott, Matthew K. Doyle, Elliott Silver, Danielle M. Brown, Chris Bashall

**Affiliations:** 1 Imaging Strategies, 30 Locke Road, Waban, MA 02468 USA; 2 Mayo Clinic IT, 200 3rd Ave SW, Pb 2-58, Rochester, MN 55905 USA; 3 Siemens Medical Solutions USA, Inc., Digital Health Services, 60 Valley Stream Parkway, Malvern, PA 19355 USA; 4 UnityPoint Health IT, 4500 Utica Ridge Road, Bettendorf, IA 52722 USA; 5 Logicalis, Inc, Healthcare Strategies Division, 9225 Priority Way, Suite 115, Indianapolis, IN 46240 USA; 6 Epic, 1979 Milky Way, Verona, WI 53593 USA; 7 McKesson Imaging and Workflow Solutions Division, 475 Allendale Road, King of Prussia, PA 19406 USA; 8 Aspirus IT, 2800 Westhill Drive, Suite 102, Wausau, WI 54401 USA; 9 Sir Charles Gairdner Hospital Radiology Dept, 1 Hospital Avenue, Nedlands, 6009 WA Australia

**Keywords:** Image exchange, Health information exchange, Medical image sharing, XDS, XDS-I, IHE, DICOM, Telehealth, Telemedicine, FHIR, Interoperability

## Abstract

The need for providers and patients to exchange and share imaging has never been more apparent, yet many organizations are only now, as a part of a larger enterprise imaging initiative, taking steps to streamline an important process that has historically been facilitated with the use of CDs or insecure methods of communication. This paper will provide an introduction to concepts and common-use cases for image exchange, outline challenges that have hindered adoption to date, and describe standards for image exchange that show increasing promise of being adopted by vendors and providers.

## Introduction

Medical imaging is one of the most costly components of patient care. Data from the American College of Radiology (ACR) indicates that diagnostic imaging accounts for 10 percent ($100 billion) of total annual healthcare costs [1]. Researchers at the Brigham and Women’s Hospital in Boston, MA [2] have estimated that a significant amount of this—nearly 9 %—is unnecessary or redundant. There is ample research demonstrating that image exchange can reduce unnecessary redundancy, and also provide other compelling value, including:

- *Cost reduction*: A New York Health Information Exchange (HIE) reduced the adjusted odds of repeat imaging by 25 % [3] by providing access to outside medical images through the HIE
- *Patient care improvements*: Not having access to outside imaging in trauma transfers can lead to significant delays in treatment [4] (up to 25 min, according to one study), which can negatively impact patient outcomes, and increase costs
- *Patient satisfaction increases*: patients involved in the RSNA Image Share Project reported an increase in both patient satisfaction and their perception of their relationship with their physician [5].

The need for providers and patients to exchange and share imaging has never been more apparent, yet many organizations are only now, as a part of a larger Enterprise Imaging Initiative [6], taking steps to streamline an important process that has historically been facilitated with the use of CDs or insecure methods of communication. This paper will provide an introduction to concepts and common-use cases for image exchange, outline challenges that have hindered adoption to date, and describe standards for image exchange that show increasing promise of being adopted by vendors and providers.

### History of Image Exchange

Since the earliest days of acquiring images of the body, having access to the images has been an intrinsic and important part of the practice of medicine. In the early days, viewing photographic films on a light box for diagnostic purposes was the task of the radiologist. However, it quickly became essential for radiologists and other clinical disciplines to discuss the images in a team setting.

A major issue for providing optimal care during the early years of film was that once the physicians left the radiology department they did not have access to the film, or the film had to be transported and tracked. An analog film can only be in one location at a time. As such, clinical care sometimes suffered due to the lack of image availability at the point of care. Films required significant manual effort to transport to another care location, and were sometimes lost or misplaced in the process or not available when they were needed. This factor led to repeat imaging examinations for the patient. Not only would the duplicative examination result in increased dose exposure for the patient, it would delay the start of medical care and add costs to the patient care process.

Fast forward to today. Since the adoption of picture archiving and communication systems (PACS) in the late nineties and early 2000s, imaging exams have been stored digitally, and film has effectively been eliminated. Yet, when patients travel from provider to provider, healthcare is still wrestling with image availability at the point of care. Patients receive digital copies of their imaging studies on CDs. When patients travel to another hospital, they bring their CDs with them. Although CDs are easier to transport than bulky film folders, the risk still exists that this fragile media could be damaged and become unreadable. CDs can also be easily misplaced or lost by the patient, and some patients simply forget to bring their CDs to the appointment. Further, each CD burner manufacturer created their own approach to storing the images, reports, and image viewer on the CD—no two are exactly alike. At the receiving end, the images are sometimes viewed using the viewer on the CD, but more commonly copied from the CD, updated with local patient and order information, and loaded into the local PACS. This requires a significant manual effort in order to get the outside images into the radiologists familiar local environment and tools [7].

If the CD was bad or damaged in some way, it may not be not possible to view or retrieve all of the images. And rarely are the reports available with the images. This creates dissatisfaction for the viewing provider. Despite the significant progress, it is clear that image-sharing practices need to be modernized to take advantage of the electronic exchange of images with related reports.

Health systems should take the opportunity to view image exchange technology as more than a way to streamline dealing with CDs. Electronic exchange provides opportunities for improved operational workflows that can positively impact patient care, reduce cost, improve patient and clinician satisfaction [8], and can even increase revenue opportunities in key service lines. While CDs will continue to be used for some time as a way to exchange images, numerous vendors provide robust—though proprietary—image exchange solutions. While this proprietary approach is less than optimal for ubiquitous exchange, it has provided a vast improvement over CD-based exchange, and laid an important foundation to support the emerging next generation of interoperable, standards-based image exchange [9].

### Image Sharing Use Cases

We will examine image exchange through the lens of three common-use cases, describing the workflow of each in a generalized way, and noting key business value and patient care improvement opportunities that can result from the use of electronic exchange. Appendix A includes a more extensive list of use cases. The benefits described for the three common-use cases are also relevant and applicable across many of the other use cases listed in Appendix A.

It is worth noting that the use cases and workflows described below are based on the use of software that is commercially available today. They do not necessarily utilize the standards-based exchange technologies described later in this paper, though use of such standards could further streamline these workflows.

### First Common-Use Case: Emergency Consult/Transfer

Approximately 50 % of trauma patients receive at least one CT at a referring facility before being transferred [10]. When patients are transferred for emergency care without sending their imaging exams in advance, clinical staff at the receiving site have limited information about the patient. This lack of information makes it difficult to conduct advance care planning, or to provide remote consults about the care needs or urgency of a patient transfer. Electronic image exchange enables remote consults for potential transfer patients, and allows the receiving care team to provide transport guidance, and to do advance planning prior to the patient’s arrival. It also reduces unnecessary transfers: a study at the University of New Mexico Hospital’s Level I trauma service demonstrated that allowing consulting doctors to see images before a patient transfer allowed them to avoid more than 40 % of potential transfers [11].

Let us assume that the facilities in this use case have an existing relationship, and have a history of electronic image exchange, and let us explore the value that electronic image exchange brings to emergency transfer care.

- At 2 a.m. a community hospital receives a patient with a head injury related to a motor vehicle accident. They perform a CT scan on the patient, but they do not have a neuroradiologist available or on-call, and need specialty advice for this patient’s care. They reach out to the regional tertiary care facility. Following the agreed potential patient transfer protocol, the ED physician at the community hospital places a call to the tertiary facility’s patient transport center and reaches a transport nurse.
- The doctor and the nurse discuss the patient, and the transport nurse asks the ED physician to send the CT scan. The radiology technologist (or the ED physician) sends the exam from PACS via an electronic exchange service between the community hospital and the tertiary care facility. This task is completed within a few minutes.
- The transport nurse is notified that the exam has been sent. The exam is automatically downloaded to the local PACS environment at the Tertiary Care facility, where they do a QC check, and make it available to the local neuroradiologist. The exam might also be made available via a local image exchange solution or even via a mobile app from the image-exchange cloud.
- The neuroradiologist accesses the exam and calls the community hospital ED physician to discuss the patient. Several possible clinical scenarios can result. Three are described in Table 1, along with descriptions of the patient impact, and the business benefit of the electronic image exchange provided.

**Table 1:** Three image exchange scenarios

Scenarios	Patient and clinician impact	Business benefit
1. The patient’s images indicate no severe trauma. Patient has a concussion, which can be effectively managed at the community hospital. Patient does not transfer.	•Community hospital physicians can make complex treatment decisions confidently. •Appropriate patient care plan is put in place faster, and the patient is discharged sooner. •Patient is not transferred unnecessarily—reducing stress, and inconvenience for family.	•Unnecessary transfer costs are avoided. •The tertiary facility’s ED saves a bay for a more seriously ill or injured patient. •Total patient cost of care is a fraction of what it would be if the patient were transferred. •The consulting service provided by the tertiary care facility will likely encourage the community hospital to build a stronger referral relationship with the tertiary care facility.
2. The patient’s images suggest a significant edema requiring immediate surgery. A helicopter transport to the tertiary care trauma center is arranged and the patient is transferred. The trauma team at the tertiary care facility is assembled, and an OR is prepared.	•New patient imaging may not need to be performed upon arrival of the patient. The receiving team can prepare using the existing images. Such timely action can improve the patient outcome. •The trauma team is able to make care decisions and prepare for surgery in a less hurried manner, potentially reducing clinical errors. •Trauma and surgical staff both have access to these outside images, enabling them to collaborate on patient care planning from their respective locations.	•The total cost of care for this patient, including rehabilitation time, is reduced. •Unreimbursed repeat CT is likely avoided. [12]
3. The patient’s images indicate that the injury is so severe that the patient will expire soon, or during transit. The physicians discuss options, and decide not to transport the patient, but to instead provide comfort measures at the community hospital.	•Community hospital physician can make treatment decisions confidently. •Patient’s family is not given false hope of recovery, nor are they forced to travel needlessly. And, they receive reassurance that appropriate specialists have consulted on the injury.	•Cost of patient care is reduced. •The tertiary care facility does not incur the costs of a patient that they cannot treat.

### Second Common-Use Case: Telehealth (Tele-burn, Wound Management)

Telehealth care of a patient with a severe burn presents two image exchange variations: provider-to-provider, and patient-to-provider. In the provider-to-provider example, an EMS team may need advice to evaluate the severity of a patient’s burn-related injuries to determine where to transfer the patient. In the patient-to-provider example, a patient may be able to reduce the number of times they travel to a hospital for check-ups to evaluate how a burn wound is healing. Without a secure image exchange solution, these use cases might happen using non-HIPAA-compliant smart phone photo sharing, or might not happen at all.

### Tele-Burn Evaluation

**Fig. 1:**
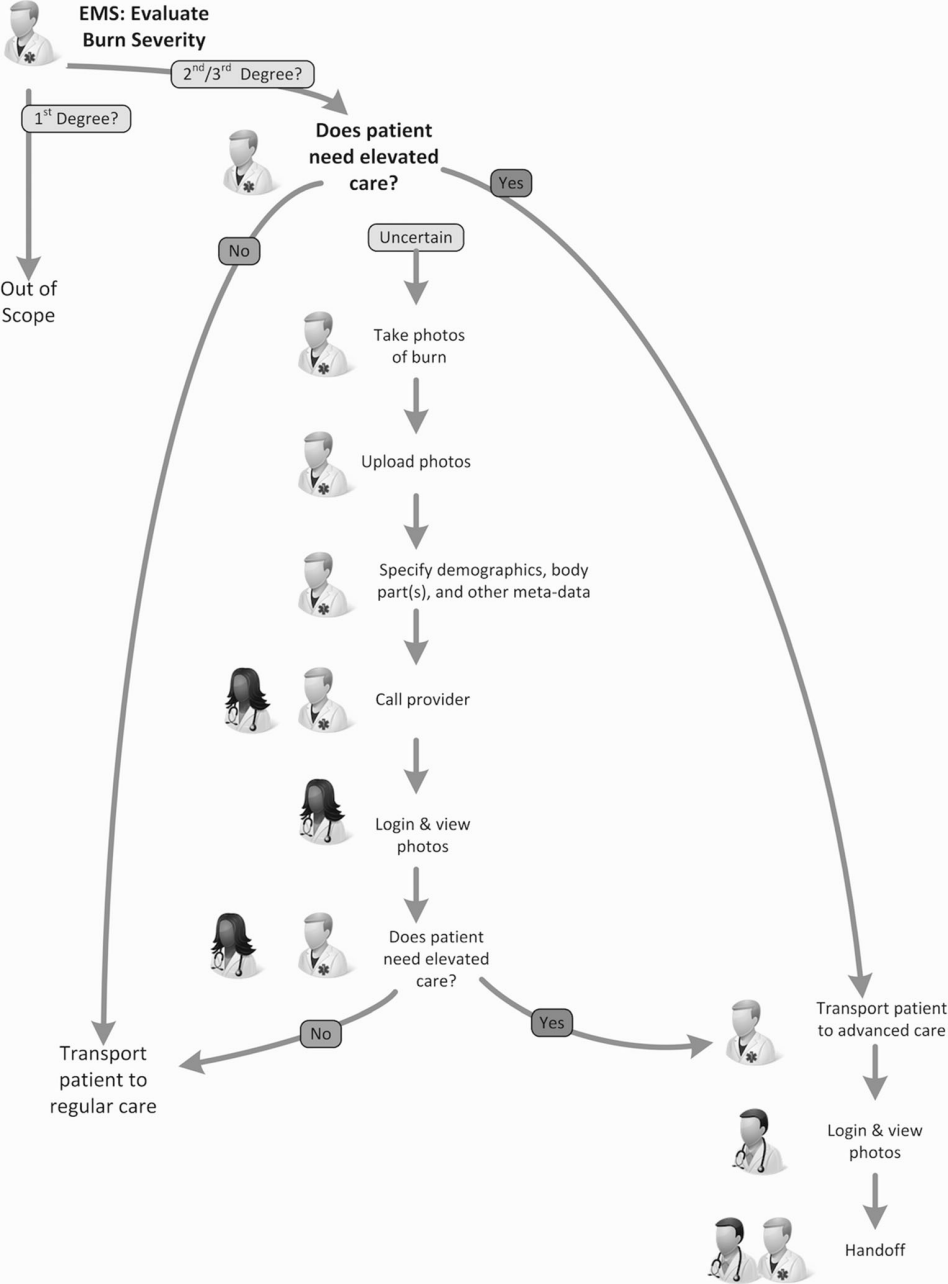
Graphical view of the workflow for the tele-burn use case for transport and care evaluation.

In the EMS use case, the workflow is very similar to the emergency consult use case described earlier. The exception is that rather than the radiology technologist pushing DICOM exams from the PACS, the EMS team uses a HIPAA-compliant photo application to take a picture of the burn to send it to the tertiary care facility. As with the emergency consult use case, the availability of an expert consult can reduce unnecessary transfers to the tertiary care facility and expedite necessary ones. It can also reduce total cost of care for a patient. See Fig. 1.

### Remote Wound Management Monitoring

In the patient-to-provider example, let us assume that the remote wound care monitoring workflow is embedded within the patient portal, and that the patient is able to use a smart phone to upload a photograph to their account. The 30-year-old patient is technology-savvy and has a history of clinical compliance, but she lives in a rural area with poor access to transportation. She and her primary care physician agree that the burn is healing, but that additional monitoring is still necessary. Her transportation issues prevent her from making frequent trips to the clinic. The physician requests that the patient use a patient portal to send a daily photograph of the wound. Each day, the patient takes a photograph of the wound, and uploads it through the patient portal. The physician (or a member of her staff) reviews the images, and sends a progress note message to the patient daily. After several days, the wound has healed sufficiently to terminate this monitoring. The patient’s experience is greatly improved. She has saved hours of travel time, has not missed any consults, and has paid fewer co-pays. The clinician has spent significantly less time with the patient, while also ensuring that the wound is appropriately monitored and infection is avoided. As more health systems shift from fee-for-service to more value-based capitated models, more states and payers are beginning to reimburse clinicians for providing such telehealth visits [13]. As a result, it is increasingly likely that the clinician is compensated for these telehealth visits.

### Third Common-Use Case: Scheduled Outpatient Encounter

The wide range of image exchange uses possible in scheduled outpatient encounters is illustrated through the following scenarios: a request for a second opinion, and two variations on preparation for a visit.

### Patient with Breast Cancer Diagnosis Seeks a Second Opinion

Following a diagnosis of breast cancer, the patient determines that she wants a second opinion. Before she leaves her physician’s office, she requests copies of her imaging exams, which she receives on a CD. Once home, she goes online and identifies two highly ranked cancer centers near her. She researches their medical staff and identifies oncologists at each of the facilities that she wants to contact for a second opinion. When she calls the first facility, the scheduler asks her if she has had prior imaging, and has access to CDs. Since she does, she is given a secure URL to upload the exams. She uploads them, and schedules an appointment for the next day. The second facility asks her to send them CDs of her exam history, and schedules an appointment for her in two weeks, to give them time to receive and review these exams. Before the scheduled appointment with the second facility happens, she cancels that appointment, as she has already been seen and started treatment at the first facility.

### Neurosurgery Clinic Obtains Exams in Advance of Patient Appointment

At the start of every week, administrative patient liaisons from this neurosurgery clinic contact patients who are to be seen the following week. They remind patients of their appointments, and ask if they have imaging that is relevant to their appointment. As in the previous breast cancer example, the staff invites patients to upload from home any exams they may have, or asks the name of the facility where relevant exams were acquired, and electronically obtains the exams on the patients’ behalf. Once the images are accessible at the neurosurgeon’s location, the neurosurgeon, or a member of the clinical staff, reviews the images. If the images are of poor quality, or inadequate for diagnosis, patients are called and scheduled for necessary, appropriate imaging the day of the neurosurgery visit. Further, if the imaging indicates that the patient condition is not a good fit for the neurosurgeon; the office can refer patients to a more appropriate specialist. During patients’ first encounter with neurosurgeons (or other specialist), there is ample clinical information available to them to have a productive, clinically focused encounter. The neurosurgeon’s calendar has more new patients, including more that are precisely aligned to the specialty.

### Rheumatologist Requests Prior Exam During the Encounter

In our rheumatology example, a patient arrives at the clinic for a consult on arthritis in his hands and wrist. The clinician discovers that the patient recently had an MRI, but did not bring it to the appointment. The clinician’s staff contacts the patient’s provider and requests that the exam be sent electronically. The images arrive during the patient visit, and the appointment continues informed by the MRI images.

As in the prior use cases, the use of electronic image exchange provides greater satisfaction and less frustration for both the patients and providers. Patients have faster access to better, more focused care, and clinicians avoid spending valuable patient care time either dealing with CDs, or in the absence of access to relevant, quality imaging.

### Image Exchange Standards

Historically, image exchange meant transporting films from one care facility to another. A paper copy of the diagnostic report might be sent with the films. Radiology departments created outside film management processes to register, track, and transport the films where they were needed, and return them to the patient or original site when done. The DICOM standard for both storing and communicating medical images exams was initially created in early 1990s, and has been well adopted by medical imaging modalities in Radiology, Cardiology, Ophthalmology, and the many departments that utilize Ultrasound imaging. The IHE (Integrating the Healthcare Enterprise) XDS (Cross-Document Sharing) and XDS-I (Cross-Document Sharing for Imaging) integration profiles leverage DICOM, HL7, and other standards to define a consistent methodology to exchange images and medical information between institutions. A brief explanation of these standards, their evolution, and use for image exchange is provided below.

### DICOM

As imaging became digital, Part 10 of the DICOM standard defined how to store images onto removable media (e.g., CD/DVD’s) and CD burners replaced film printers as the primary way to exchange images between care facilities. The *DICOM images* on the CD/DVD are carried by the patient, or mailed to the destination health care facility, where they can be loaded into their PACS to be viewed, compared, interpreted, or have additional image processing performed. But there are a number of manual steps required to burn and transport the CD/DVD, and then load the images for viewing. In the emergency transfer case the CD/DVD’s were sometimes taped to the patient for transport, and in some cases the transport itself was delayed because the images were still being burned onto the CD/DVD. If there was a diagnostic report on the CD/DVD, the report format, where it could be found on the CD/DVD, and how the correct report related to the correct imaging study, varied by CD burner vendor and particular implementation.

Improved wide area network connectivity and VPN’s (virtual private networks) have provided a secure way to transport patient health information. Hospitals and clinics that frequently send radiology images to each other would sometimes establish a VPN connection between the two sites. This enabled the DICOM images in the PACS to be directly sent using *DICOM protocols* between facilities instead of burning a CD. It usually required manual human communication, typically a fax, email, or phone call, to alert the receiving site that a study was being sent, and provide additional information, such as the diagnostic report, to go along with the images. And it usually required some work on the receiving side to load the images onto the local PACS and get the paperwork to the appropriate physician.

With the advent of cloud-based applications a new group of cloud-based image exchange vendor solutions became available. Most of these solutions provide a gateway device that can be placed at sites that want to exchange images. These gateway devices use DICOM protocols to interface with the *DICOM sources* at each site (e.g., VNA, PACS, or DICOM modalities) and upload the images to the vendor cloud. With routing rules implemented in the vendor cloud, and the proper configuration between the gateway devices and the destination site PACS, it is usually possible to exchange DICOM images directly between the two sites local systems—with little or no manual effort. Some of the vended solutions provide additional logic to map the patient identifiers (which are sometimes different between the exchanging sites) and other interface logic that might be needed, to exchange related reports, or place orders into the receiving sites systems. This solution provides a significant improvement over both CDs and the point-to-point VPN connection exchange methods, but still has the limitation that it is proprietary, and requires that the sending and receiving sites use the same vendor software and/or gateways. If two sites that want to share have different vendor image exchange solutions installed, they are generally not able to easily exchange images, or it requires extra manual steps in order to do so.

Each of the digital image exchange solutions described above relies on *standards*-*based DICOM images*, *standards*-*based DICOM interfaces*, and/or *the DICOM removable media standard*. And each solution can successfully exchange the original DICOM images so they can be used for patient care at the remote site. While each phase of the evolution of the digital image exchange solutions described above provides a better solution with less manual work, each solution still has limitations or drawbacks. The CD/DVD’s require manual effort to burn and transport and are not convenient at the receiving end. The VPN is a dedicated point-to-point connection, requires a manual phone call, or fax or email, and usually some other manual steps. The cloud-based solution requires the same brand of vendor gateway to be at each exchanging site. And there is no consistency to if, and how, the diagnostic report accompanies the images.

### Document and Image Sharing IHE Integration Profiles (XDS, and XDS-I)

IHE (Integrating the Healthcare Enterprise) is an initiative to improve how computer systems exchange medical information, including images and reports. IHE is not a standard, but uses established standards, like DICOM and HL7, to accomplish specific medical workflows. IHE calls these medical workflows “integration profiles.” The idea is that a given medical image workflow, like sharing images with reports, might involve multiple systems, standards, and interfaces that work together to accomplish that workflow, or integration profile.

Each integration profile is made up of the set of actors and transactions. IHE defines the specific transactions (using standards-based interfaces) that an actor must support, and the information (fields) that need to be present in those interfaces. This level of workflow-driven interface specification helps ensure compatibility across health care environments that may use different vendor products.

Two IHE integration profiles are of particular interest for exchanging images with reports.

XDS (Cross-Enterprise Document Sharing) is an IHE integration profile for sharing medical record documents with other health care providers. These documents could be radiology reports, lab results, clinical notes, CDAs, or a variety of other medical record documentation, including JPEG photographs.

XDS-I (Cross-Enterprise Document Sharing for Imaging) is an IHE integration profile that extends XDS to include the sharing of DICOM images, presentation states, key image notes, and other related imaging content.

The approach used by XDS and XDS-I is not a point-to-point “push”. Rather it is a “push/pull”. A group of hospitals and clinics that want to share images together form what IHE calls an *affinity domain*. Medical record documents or DICOM imaging studies that are eligible to be shared in that affinity domain are registered into a central *XDS registry* that is shared by all the participating clinics and hospitals. A copy of the documents is stored to one or more *XDS document repositories* accessible by all participating clinics and hospitals. With XDS-I, the DICOM images usually remain in each local or regional *XDS imaging document source* (usually a PACS or VNA). For each imaging study, an imaging manifest document is created that describes the image content included in that study. The imaging manifest is saved as a document in the XDS document repository and indexed in the XDS registry, just like any other document.

**Fig. 2:**
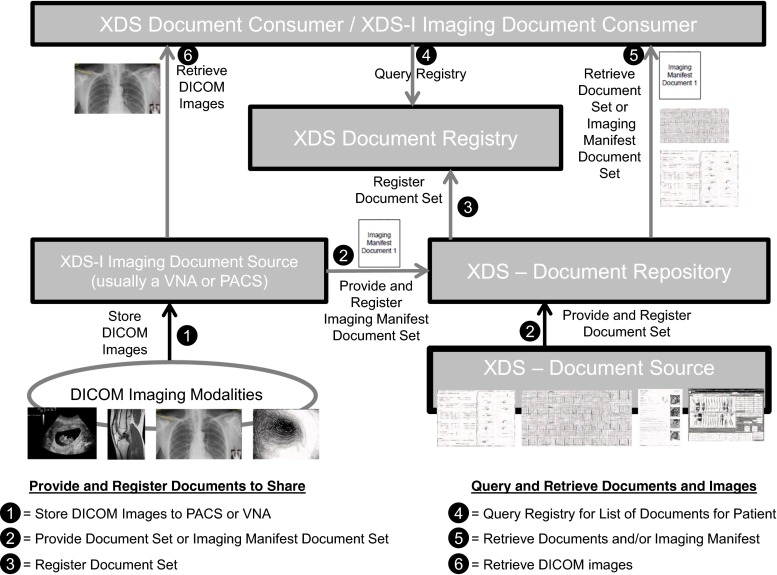
Shows how documents and images are shared between sources and consumers using actors and transactions based on the XDS/XDS-I IHE Integration Profiles. Documents are stored in an XDS document repository and registered. DICOM images are stored in an Imaging Document Source, and an Imaging Manifest Document is created, stored in an XDS document repository and registered.

To retrieve documents or images, a consumer application queries the registry to get a list of documents available for a given patient in the affinity domain. The consumer can then retrieve the desired documents from the appropriate XDS document repository. If the retrieved document is an imaging manifest, the manifest provides the information necessary to retrieve the DICOM images from the appropriate XDS imaging document source (see Fig. 2).

Several other IHE integration profiles (PIX, XCPD, and XCA/XCA-I) can be used with the XDS and XDS-I implementations. The PIX (Patient Identifier Cross Reference) provides the integration to map and utilize the different patient identifiers that a patient may have in a given affinity domain. The XCPD (Cross-Community Patient Discovery) integration profile also provides patient matching, but uses a demographics-based heuristic method to determine the patient. XCA (Cross-Community Access) enables two different XDS affinity domains to “connect together” and exchange documents. XCA-I (Cross-Community Access for Imaging) enables to different XDS-I affinity domains to “connect together” and exchange DICOM images.

The XDS integration profile has been widely adopted both within and outside of the USA. The XDS-I integration profile is commonly used outside the USA. A joint image-share validation initiative was announced at the 2015 RSNA (Radiological Society of North America) meeting. This initiative, between RSNA and the Sequoia project, will certify compliance of image exchange vendor products to specific IHE actor/transactions in the XDS-I, and XCA-I integration profiles. This program will encourage vendor compliance and movement toward increased use of the XDS-I and XCA-I integration profiles to enable the exchange of DICOM images.

### FHIR and DICOM Web—Standard Web Services

New “standards-based” RESTful web services are available which hold the promise to fuel the next generation of secure healthcare image and information exchange solutions in both traditional web-based and mobile environments. FHIR (Fast Healthcare Interoperability Resource) provides a new framework created by the HL7 standards body. The DICOM standards body is providing DICOMWeb, the RESTful standards-based web service specifications for the next generation of DICOM communications. IHE has created new integration profiles, MHD (Mobile Access to Health Documents) and MHD-I (Mobile Access to Health Documents for Imaging) that utilize the FHIR and DICOMWeb interfaces to augment the XDS and XDS-I integration profiles.

### Image Exchange Challenges

While electronic image exchange provides abundant opportunities for patient care and business workflow improvements throughout an organization, health systems sometimes achieve only limited success. It may be that they do not reach high volumes of relevant exchange in important service lines, they may only introduce exchange in a few areas, or they do not craft effective, scalable workflows for exchange. We will describe some issues that cause organizations to falter, and suggest ideas to consider that can help avoid such pitfalls should your organization begin an image exchange project.

### Governance, and Radiology and IT Collaboration

Image exchange use cases lie at the cross section of radiology and other image-focused service lines and may highlight the competing needs these groups may have. For instance, radiology may feel responsible to provide secondary reads for all exams that are sent to PACS, and have concerns about their capacity to take on additional secondary reading responsibilities if high volumes of outside exams are sent. As a result, they may work to limit the number of outside exams that arrive in PACS. Meanwhile, Neurology, Oncology, and other service lines require the ability to view and to compare these outside images to new ones as they treat referred patients over time. They may also need expert specialty opinions from their subspecialty radiology colleagues. Or, radiology may choose to centrally manage an enterprise exchange solution they have already licensed, and inadvertently prevent or delay other departments from benefitting from it fully.

These issues reflect a lack of clear, cohesive organizational goals for image exchange. They are precisely the types of conflicts that can cause low adoption of image exchange or may even cause service lines to invest in solutions independently, for use only by their own departments. This further complicates an enterprise’s efforts. An effective governance organization will clarify the enterprise’s strategic goals, deliver clear communication to stakeholders throughout the organization, provide a framework for decision-making about image exchange, and can help arbitrate conflicts that arise.

Most health systems, as a result of other initiatives such as implementation of an enterprise-wide EHR rollout, already have some sort of enterprise governance structure in place for managing large new initiatives. But, because imaging-related technologies (including modalities and PACS systems) have traditionally been managed and operated within a radiology IT structure, imaging may not be included in existing enterprise governance models. The models may be sound, but may not yet be applied to imaging-related projects, and may be missing representation from imaging-focused specialties. Expanding the scope of existing governance models to consider enterprise imaging needs, including exchange, is advisable. Adding other leaders or clinicians from imaging-intensive areas to this governance structure can balance disparate objectives of clinicians, IT, and business leaders. See an associated whitepaper from this series, Enterprise Imaging Governance: Needs, Models, and Intents To Consider to learn more [14].

### Communication and Engagement Strategy for Inter-facility Exchange

Successful image exchange deployments leverage functions including marketing, partner outreach, and education. These are vital teams for helping to focus the organization’s strategy and efforts for community outreach to ensure adoption of inter-facility exchange with key strategic referring facilities. Health systems sometimes leave this communication responsibility in the hands of PACS administrators, or other team members. These staff members may not have the skill set or contacts to forge these strategic relationships outside of the organization. A multi-level approach to this outreach, including clinician-to-clinician and administrator-to-administrator communication can open a channel for exchange, which can then be supported by PACS administrators and educational resources.

### Vendor Interoperability (or Lack Thereof)

Another stumbling block, outside the control of the organization, is that many current image exchange solutions are not yet standards-based or interoperable. As a result, these solutions do not “naturally” communicate with each other. Some of the facilities with whom your organization needs to exchange exams may have different vendor solutions in place and may not be interested in installing your vendor’s solution to do the same thing as the one they already have installed. Imagine if you had to carry an AT&T phone to talk to your friends that have AT&T, and a Verizon phone to talk to your friends that have a Verizon phone, and so on. This is the current state of image exchange technology today, and your organization will need to develop an exchange workflow that accommodates this. You may need multiple methods for moving exams from one health system to another, but you should be able to develop a consistent approach and rules for patient identification and procedure labeling.

Many recent developments and announcements, among them ONC’s Shared Nationwide Interoperability Roadmap [15], and the Interoperability Pledge [16] (which many IT vendors, healthcare systems, and provider organizations have taken, in which they commit to improve consumer access, to avoid data blocking, and to adopt IT exchange standards) suggest that the government is working to foster vendor interoperability, and that vendors may be beginning to respond to buyer and government pressure for real interoperability. And a number of public-private, multi-stakeholder collaborative efforts, such as Carequality and the RSNA Image Share Validation Project are beginning to foster tangible progress toward interoperability.

But large-scale results remain to be seen and pressure needs to continue. During a vendor selection process, we strongly advise scrutinizing the vendors’ commitment to standards-based exchange, and require that they commit to a delivery roadmap and timeline for providing these tools. (We encourage having similar conversations for any incumbent vendors, as well, as these existing systems will need to communicate with any new vendors.)

### Integrating Outside Exams with Local Systems

Organizations may also wrestle with how to integrate outside images into the existing local environment, tools, and workflows. Managing exams with CD-based workflows is often a manual back office function, triggered through paper forms and CDs in interoffice envelopes. Real value in image exchange comes from transforming this into a proactive service that makes the outside images available to the appropriate departments in their familiar local tools (EMR, PACS, VNA, Enterprise Clinical Image Viewer, etc.) with minimal manual effort.

In order to accomplish this, it is important to develop the criteria and processes for how and when outside images, and related information will be made available and managed in local systems. These criteria and processes will be based on answers to questions such as:

### How Will Patients Be Positively Identified?

This is an important challenge and should be carefully considered to ensure a patient’s images are never associated with the wrong patient in the EHR. You will need to determine rules for which data elements will require an exact match between the DICOM exam or other patient identifying information and the EHR data, and which classes of users can, and what tool is used to, override a “failed exact match” and associate an outside exam to a patient (for instance, if a patients outside exams have a different spelling of last name). And you will need to determine workflow—specifically, when will a registration be created and who is responsible for creating it. Organizations may have already considered this question for other reasons (Meaningful Use transitions of care, medical records scanning, image import from a CD, etc.) and may be able to reuse or adapt the existing process to work for image exchange.

### What Local Systems Will the Outside Exams and Related Workflow Be Available in?

Governance input will help determine when outside exams should be loaded into the local VNA and PACS, and as a result, when will they be available in the Enterprise Clinical Image Viewer and EMR. If exams are sent to the local PACS, you will need to define how and when orders should be created and structured. And, a vital decision that must be made is how the image exchange solution integrates with the EHR driven workflows—for instance—can clinicians request or push images from within the EHR.

### Who Is Responsible for Certain Activities?

Determining who performs certain tasks and workflows is as important to a project’s success as defining the processes themselves. Clarity is needed on the questions of what type of staff will be responsible to reach out to patients or outside facilities to obtain exams and what are the right marketing and education programs to educate referring and transfer facilities about the exchange services. Additionally, you will need to define who will be responsible for troubleshooting issues or performing image transfers during normal business hours and during off hours.

You will need a granular breakdown on what roles are allowed to request that exams be sent to other facilities, and which roles actually send the exams. Will received images be triaged or evaluated for appropriateness before they are made available to the reviewing physician? If yes, who will be responsible for the triage function? How will physicians be notified that images are available for review? Thoughtfully considering questions such as these will contribute to project success.

There are a few other key questions that require organizational policy decisions to be made, as they can have a significant impact on budgets, medical legal liability, and radiologist staffing. They relate to the responsibility and policies the organization takes on when they incorporate outside exams into their systems and workflows.

### What Is the Policy on Keeping Outside Exams in Local System Archives?

A key question to be answered is how long outside exams are maintained and available in local systems. Will these exams be treated the same way as locally acquired exams? A factor to consider is can the same exam be reliably retrieved again from the outside source if it is needed for subsequent patient care, or is it better to keep the exam available locally. There are nuances to consider in answering these questions, such as, if a local patient care decision is made based on outside exams, or if another imaging series (e.g., 3D view) is created from the outside images, do those outside exams need to be retained locally for medico legal reasons? Can outside images stored in your local system be exchanged with other outside institutions? Additionally, your organization needs to consider how to prevent treating your own local images as outside images if they are mirrored back to you (e.g., brought in by the patient on a CD, or sent in electronically). While guidance exists to understand base requirements and support making these decisions [17], state requirements, clinical interests, and health system policies must all be considered.

### What Is the Policy of Providing Secondary Reads of Outside Exams?

There should be careful discussion when developing policies (and the often cumbersome, but necessary supporting workflows) [18] for providing secondary reads. Organizations need to consider when a second opinion is warranted or appropriate. There are often disagreement between interpretations of imaging studies by generalist community radiologists and specialty radiologists at tertiary care facilities. As studies show that disagreement is common, and may exist for between 7 and 30 % of certain types of exams [19–21] this question warrants serious study. There are a broad range of approaches here: some organizations overread every outside exam, and others do none. Still others will overread all ED exams, or overread no more than 2 exams per outside patient, as specifically selected by clinicians. The obvious impact here is on radiology reading resources, and your organization will need to weigh the cost of over-reading outside exams against the potential patient care improvements and expedited care, or possible legal risk avoidance as a result of having local, accurate, subspecialty radiology reads on outside patient exams.

## Conclusion

A successful image exchange program can be transformational for both care providers and patients. It can open doors to new types of services, and levels of service, and care that are not possible when images are transported on CDs. EMR and “health information exchange” initiatives need to leverage the sharing of related health information and images together (images with reports and patient history) in tools that are both convenient and familiar to the care providers. And automated integration (with minimal or no manual intervention) of outside images with local enterprise and departmental systems (VNA, PACS, Enterprise Clinical Viewer), makes the outside images available in the familiar tools departments use for their day-to-day work.

To fully realize this is an enterprise effort, much like the implementation of an EMR. It requires a good understanding of the image exchange use cases and related workflows, careful planning, with the right people involved, and strong governance and leadership.

## Appendix A

The table below lists a variety of image-sharing use cases and information regarding the urgency (of the transfer) and the type of images that are typically involved in each. Three of these use cases (Tele-Burn, Emergency Transfer, Second opinion) were described in the paper in greater depth.

**Table:**

Image-sharing use case group	Image-sharing use case name	Urgency	Radiology	Cardiology	Photograph	Live video	Other imaging
Telemedicine							
	Tele-burn	High	No	No	Yes	Maybe	No
	Tele-stroke	High	Yes	No	No	Yes	No
	Real-time eHealth consult (physician to physician)	High	Yes	Yes	Yes	Maybe	All
	Store and forward eHealth consult (physician to physician)	Medium	Yes	Yes	Yes	No	All
	Primary interpretation services	High	Yes	Yes	No	No	All
	To support remote ICU tele-presence	High	Yes	Yes	No	Yes	All
Normal care							
	Physician access to patient history and studies for comparison	High	Yes	Yes	Yes	No	All
	Patient access to their patient health record	High	Yes	Yes	Yes	No	All
Patient care at a different facility							
	Emergency transfer	High	Yes	Yes	Yes	No	All
	Emergency consult	High	Yes	Yes	Yes	Yes	Yes
	Continuity of care transfer	Medium	Yes	Yes	Yes	No	All
	Patient out of town (e.g., on vacation)	High				No	
	Patient moves	Low	Yes	Yes	Yes	No	All
	ACO or other value-based reimbursed patient imaged at out of network facility	Medium	Yes	Yes	Yes	Yes	All
	Ongoing exchange between patient medical home and tertiary care facility providing specialty treatment	Medium	Yes	Yes	Yes	Yes	All
	Ongoing exchange between hospital and site providing services the hospital does not provide (proton therapy, for instance)	Medium	Yes	Yes	Yes	No	No
	Remote organ evaluation of potential cadaver donor organs	High	Yes	Yes	Yes	No	No
Second opinion or patient referral							
	Patient travels to a different care facility for second opinion	Medium	Yes	Yes	Yes	No	All
	Physician consults a specialist for second opinion	High	Yes	Yes	Yes	Maybe	All
	Physician consults a surgeon	High	Yes	Yes	Yes	Maybe	All
	Patient requests an “electronic second opinion”	Med	Yes	Yes	Yes	No	All
	Provider–imaging center–specialist (three-way care relationship)	Med	Yes	Yes	No	No	No
	Ad hoc second opinion	Med	Yes	No	No	No	No
Collaborative treatment							
	Tumor board	Medium	Yes	Yes	Yes	Yes	All
Home care/self care							
	Patient online request with patient photo to upload	Med	No	No	Yes	Yes	No
Clinician convenience							
	On-call specialist has slow VPN access to hospital	High	Yes	Yes	No	No	No
	Trauma team member is not near home or hospital when a consult is needed—mobile access	High	Yes	No	Yes	No	No
